# Bilateral Lacrimal Gland Lymphoma - Case Presentation

**DOI:** 10.22336/rjo.2024.36

**Published:** 2024

**Authors:** Violeta Ioana Prună, Diana Mihaela Ciuc, Valeriu Gabi Dincă, Viorel Mihai Prună

**Affiliations:** * Department of Ophthalmology, CF2 Clinical Hospital, Bucharest, Romania; ** Department of ENT, “Titu Maiorescu” Faculty of Medicine, Bucharest, Romania; *** Department of Surgery, “Titu Maiorescu” Faculty of Medicine, Bucharest, Romania; **** Department of Neurosurgery, “Carol Davila” University of Medicine and Pharmacy, Bucharest, Romania

**Keywords:** lacrimal gland lymphomas, orbital tumors, MALT, B-cell lymphoma

## Abstract

Lacrimal gland lymphomas are rare orbital tumors, constituting a minor fraction of all orbital and ocular adnexal malignancies. This case study presents an 83-year-old male with bilateral lacrimal gland tumors, more prominent in the left orbit, causing decreased visual acuity, red eye, excessive tearing, and diplopia. Initial ophthalmological evaluations and imaging suggested bilateral lacrimal gland lymphoma, confirmed by histopathology as diffuse large B-cell non-Hodgkin lymphoma of the MALT type. Due to the significant tumor size and risk of visual function loss, surgical intervention was performed, followed by corticosteroid therapy. Postoperatively, a marked improvement in symptoms and a reduction in tumor size were observed. This case underscores the importance of comprehensive diagnostic approaches, including clinical, imaging, and histopathological evaluations, highlighting the need for a multidisciplinary approach in managing rare orbital tumors like lacrimal gland lymphoma. The patient’s postoperative and follow-up care included oncological management to monitor and ensure long-term disease control and patient well-being.

**Abbreviations:** RE = right eye, LE = left eye, CT = Computer tomography, MRI = Magnetic Resonance Imaging, TOD = intraocular pressure of right eye, TOS = intraocular pressure of left eye, US = ultrasound

## Introduction

Lacrimal gland lymphomas are rare orbital tumors, accounting for a small fraction of all orbital and ocular adnexal malignancies. These tumors can present with a variety of symptoms, including swelling, visual disturbances, and eye discomfort, often posing diagnostic challenges due to their overlapping features with other orbital conditions. In this article, we report the case of an 83-year-old male from a rural area, who presented with bilateral tumor masses in the superior-external area of the orbits, more pronounced on the left side. The patient’s symptoms included decreased visual acuity, red eye, excessive tearing, and diplopia. Clinical and imaging evaluations suggested a diagnosis of bilateral lacrimal gland lymphoma, later confirmed through histopathological examination. This case underscored the importance of comprehensive diagnostic approaches and the need for multidisciplinary collaboration to manage rare orbital tumors and achieve optimal patient outcomes.

## Case report

We present the case of an 83-year-old male patient from a rural area, who presented with a large tumor mass located bilaterally in the superior-external area of the orbit, with a mass effect, especially at the level of the left orbit, causing decreased visual acuity, red eye, excessive tearing, and diplopia. The swelling appeared approximately 12 months before, but the patient described an accelerated increase in size over the past 3 months. The patient was pseudophakic and had no significant personal or family medical history. Three months before presenting to our service, the patient was examined in an ophthalmology clinic, and the examination results indicated a diagnosis of bilateral posterior chamber pseudophakia, with no mention of the orbit’s condition. At that time, the best corrected visual acuity was described as 1.0 (decimal) in the RE and 0.9 (decimal) in the LE, with normal intraocular pressures (17,0 mm Hg in the RE and 16,0 mm Hg in the LE), and refractometry readings of + 0,50 - 1,50 x 80 for the RE and - 0,5 - 1,00 x 150 for the LE. The patient was sent home with a recommendation for treatment with artificial tears.

Upon admission to our service, the ophthalmologic examination revealed: BCVA - RE = 1,0 (decimal); BCVA - LE = 0,3 (decimal), TOD = 29,0 mm Hg, TOS = 37,0 mm Hg. RE - a palpable tumor mass in the superior-external angle of the orbit, firm in consistency, non-painful, not causing a mass effect, and not interfering with ocular movement, but extending subpalpebral and under the orbital rim towards the center. Biomicroscopic examination showed marked violaceous conjunctival congestion and chemosis in the superior-temporal sector. Moreover, the following were also observed: clear cornea, a medium-depth anterior chamber with clear content, round, central, reactive pupil, and pseudophakic lens present in the posterior chamber. The fundus of the eye appeared relatively normal, with a cup/disc ratio of 0.2, but with some choroidal folds.

**Fig. 1 A, B:**
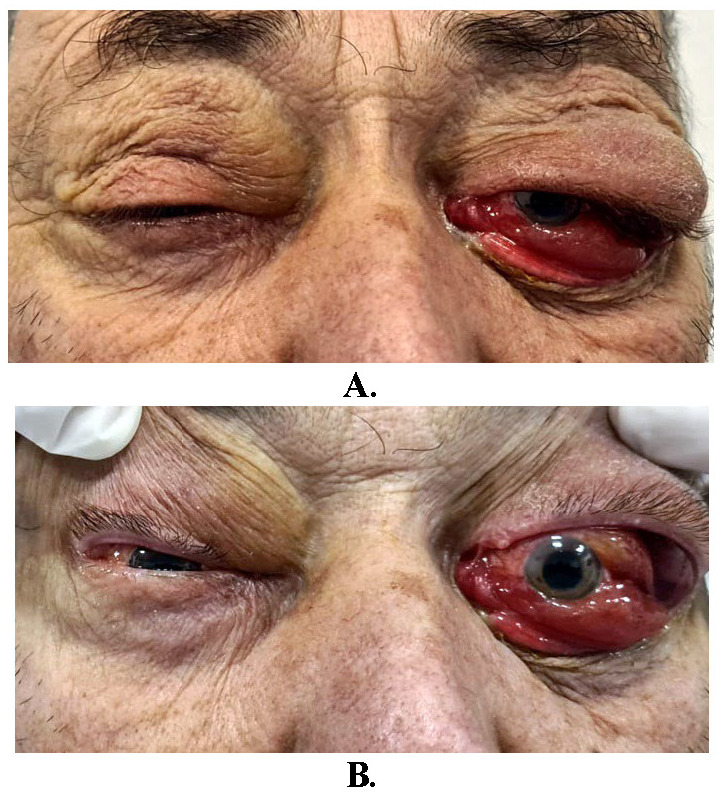
Bilateral tumoral masses in the superolateral aspects of the orbits

LE - large tumor mass (approximately 4 / 6 cm), firm in consistency, located in the superior-external area of the orbit, poorly defined, causing a mass effect, with inferonasal displacement of the eyeball and protrusion, leading to massive chemosis that prevented eyelid closure. Biomicroscopic examination revealed corneal erosions and opacities in the inferotemporal sector, a medium-depth anterior chamber with clear content, round, central, reactive pupil, and pseudophakic lens in the posterior chamber. The fundus of the eye was difficult to observe, showing a cup/disc ratio of 0.2 and choroidal folds. The patient complained of binocular diplopia due to the dislocation of the left eyeball. The preoperative images are shown in **Fig. 1 A, B**.

**Fig. 2:**
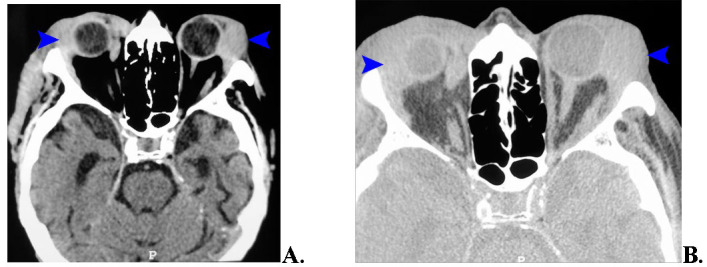
**A.** The axial image shows bilateral, homogeneous, relatively well-defined formations located superolateral to the eyeballs (blue arrows). **B.** Axial CT brain image - bone window - the tumor formations (blue arrow) do not cause erosion of the orbital walls

The CT scan showed a tumor formation in the superior-external aspect of the orbit, bilaterally, larger on the left, with posterior extension, impacting the eyeballs and the left external rectus muscle, but not causing bone erosion (**Fig. 2 A, B**).

**Fig. 3:**
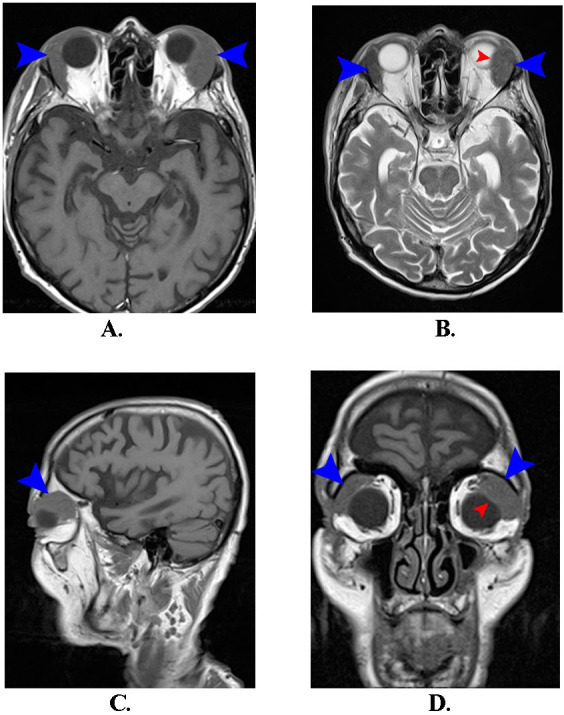
**A.** Axial T1; **B.** Axial T2; **C.** Sagittal T1; **D.** Coronal T1: reveals two tumoral masses located in the superior-external quadrants of the orbits (blue arrows). The left tumor formation compresses and deforms the left eyeball (red arrow)

The brain MRI examination showed two oval-shaped hypointense tumor formations in T1 and T2, located superolateral to the eyeballs. The tumor formation on the left was larger than the one on the right and compressed the eyeball, deforming it and displacing it inferonasally (**Fig. 3 A-D**).

**Fig. 4:**
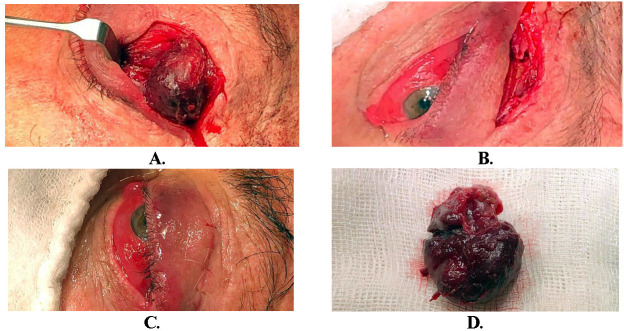
Intraoperative images. **A.** Highlighting the tumor; **B, C** Images at the end of surgery; **D.** excised lacrimal gland tumor

Following the clinical and imaging examinations, a presumptive diagnosis of bilateral lacrimal gland lymphoma was made. Inflammatory conditions of the lacrimal glands were considered in the differential diagnosis, being usually unilateral and associated with other signs of inflammation (pain, redness, etc.), as well as other tumors of the lacrimal glands, which can present similarly clinically, but are also usually unilateral. From an imaging perspective, lacrimal gland lymphomas can share characteristics with other lacrimal gland tumors, such as hyperdensity on CT and hypointensity on MRI compared to surrounding tissues. However, the bilateral nature and absence of other specific imaging features, such as calcifications or bone erosions, pointed towards the lymphoma diagnosis. Laboratory tests and additional imaging investigations did not reveal the presence of other lymphomas in other parts of the patient’s body, so the tumor was considered a primary lymphoma of the lacrimal glands. Although the therapeutic protocol in such cases initially recommends a biopsy, considering the large volume of the tumor on the left side, with impairment of eyelid occlusion and the risk of losing visual function, surgical intervention was performed under general anesthesia (**Fig. 4 A-D**). The tumor was completely removed through an anterior transpalpebral approach. The dissection was meticulously undergone, with special care taken around the external rectus muscle, which was edematous and sinuous. The excised specimen measured approximately 45 / 40 / 30 mm. In the immediate postoperative period, corticosteroid therapy was initiated, and both intra- and postoperative evolution were favorable. Corticosteroid therapy markedly reduced the chemosis and the tumor mass in the unoperated right eye, as observed in the pre- and postoperative photographs. Diplopia disappeared, corneal erosions and infiltrates began to heal, and intraocular pressures started to normalize, confirming the preoperative suspicion that the cause of the increased intraocular pressure was extrinsic, due to tumor compression.

**Fig. 5:**
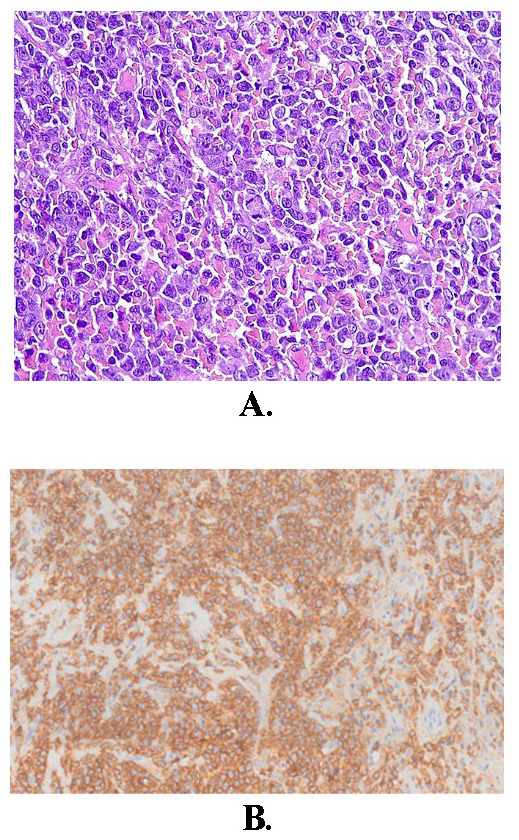
**A.** Diffuse proliferation of centrocyte-like cells, monocytosis, with rare centroblast-like and immunoblastic cells (HE staining) (ob 40x). **B.** - immunohistochemistry: CD20 (pan B) diffusely positive (objective 10x)

The histopathological examination found a diffuse large B-cell non-Hodgkin lymphoma. Immunohistochemistry confirmed a low-grade B-cell lymphoma of the MALT (mucosa-associated lymphoid tissue) type (marginal zone) (**Fig. 5 A, B**).

**Fig. 6:**
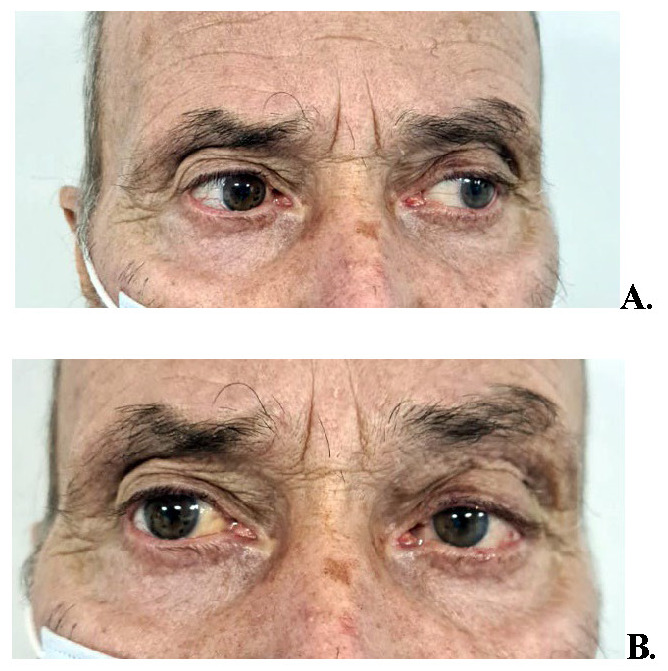
**A.** One-month postoperative shows normal left abduction. **B.** one-month postoperative shows resolution of tumors

The patient returned for a follow-up one month postoperatively, with a marked remission of the tumor masses, as seen in the photographs (**Fig. 6 A, B**). Subsequently, he was taken under oncological care.

## Discussions

The epidemiology of lacrimal gland lymphomas is characterized by its rarity, demographic variations, and histological diversity, necessitating a multidisciplinary approach to surveillance, diagnosis, and management [**1**]. The lacrimal glands tumoral pathology accounts for approximately 10% of orbital expansive processes [**1**]. Over half of the lacrimal gland tumors have an epithelial origin, one-third are lymphoid, and the remaining 10-15% have mesenchymal origin and are secondary tumors [**1**].

Epidemiological studies have revealed lacrimal gland lymphomas to be uncommon, accounting for a small fraction of orbital and ocular adnexal tumors, with incidence rates varying across geographic regions and populations [**2**,**3**]. Among lacrimal gland malignancies, lymphomas constitute a significant proportion, with varying histological subtypes including extranodal marginal zone B-cell lymphoma of MALT type, diffuse large B-cell lymphoma, and lymphoplasmacytic lymphoma [**4**-**6**].

Demographic characteristics of patients with lacrimal gland lymphomas typically include middle-aged to elderly individuals, even though cases have been reported across all age groups, with a slight female predominance observed in some series [**2**].

Environmental and occupational factors have been proposed as potential etiological contributors to lacrimal gland lymphomas, even though conclusive evidence remains limited, necessitating further research to elucidate the underlying risk factors and pathogenesis [**2**,**3**].

Challenges in accurately capturing the epidemiology of lacrimal gland lymphomas include underreporting, misclassification, and variability in diagnostic criteria and coding practices, emphasizing the need for standardized approaches and collaborative research initiatives [**2**,**7**].

The clinical picture is characterized by specific symptoms and signs of lacrimal gland conditions (swelling in the superolateral aspect of the orbit, deformation of the upper eyelid, ocular discomfort), to which the increasingly pronounced mass effect produced by the tumor is added, depending on the volume and location of the tumor mass (more at the level of the eyelid portion or, more at the level of the orbital portion of the lacrimal gland): displacement of the eyeball, involvement of the extrinsic musculature, leading to consequent diplopia; pain is not a commonly reported phenomenon by patients [**1**,**5**,**6**,**8**]. Indeed, our patient presented with the same symptoms, but, in his case, the situation was more severe, due to marked chemosis at the level of the left eye.

Systemic evaluation, including laboratory tests, bone marrow biopsy, and imaging studies of distant sites, is crucial for staging and assessing the extent of disease involvement, guiding treatment decisions, and prognostication [**2**,**4**,**8**].

Challenges in the clinical assessment of lacrimal gland lymphomas include their rarity, variable presentation, and potential overlap with other orbital and adnexal pathologies, highlighting the importance of multidisciplinary collaboration and expertise in achieving accurate diagnosis and optimal patient care [**3**,**7**].

Imaging studies, CT and MRI, have an important role in characterizing lacrimal gland lesions, providing valuable information regarding size, shape, tissue density, and involvement of adjacent structures, and aiding in preoperative planning and differential diagnosis [**9**]. CT and MRI may reveal characteristic imaging features of lacrimal gland lymphomas, including well-defined homogeneous masses with moderate enhancement on contrast-enhanced sequences, even though variability exists depending on the histological subtype and degree of vascularity [**9**,**10**].

CT scans can reveal the size, location, and density of lacrimal gland lesions, and their relationship to adjacent structures such as the orbit and surrounding bone. The appearance of lacrimal gland lymphoma on a computer tomography (CT) scan typically presents as a well-defined mass within the lacrimal gland fossa, without invasion of adjacent tissues [**1**,**9**]. MRI has better soft tissue contrast and multiplanar imaging capabilities, facilitating the assessment of lacrimal gland lymphomas concerning the globe, optic nerve, and extraocular muscles, as well as detecting intracranial extension or orbital invasion [**9**]. On MRI, lacrimal gland lymphoma typically appears as a well-defined mass within the lacrimal gland fossa. The signal intensity of the mass on T1-weighted images is usually intermediate to slightly hypointense compared to the surrounding normal lacrimal gland tissue. On T2-weighted images, the mass tends to be hyperintense, although this can vary depending on the histologic subtype, and the necrosis presence or cystic changes.

Ultrasonography (US) may complement cross-sectional imaging modalities in the lacrimal gland lesions evaluation, providing real-time assessment of tumor vascularity, cystic components, and associated orbital pathology, even though its utility may be limited by operator dependency and anatomical constraints [**9**].

Comparative studies have highlighted similarities in imaging features between lacrimal gland lymphomas and other orbital malignancies, such as adenoid cystic carcinoma and lacrimal gland adenocarcinomas, underscoring the importance of histopathological confirmation for definitive diagnosis [**7**,**9**].

Differential diagnosis of lacrimal gland masses encompasses a broad spectrum of benign and malignant entities, including inflammatory pseudotumors, dacryoadenitis, and lacrimal gland adenocarcinomas, necessitating comprehensive evaluation, and histopathological confirmation [**9**,**11**]. Clinical evaluation of lacrimal gland masses often involves distinguishing between benign and malignant etiologies, with lymphomas representing a subset of primary malignant tumors arising in this location [**9**].

Clinical presentation and patient demographics may offer additional clues for differential diagnosis, with lacrimal gland lymphomas often presenting as painless, slowly progressive masses in middle-aged to elderly individuals while infectious or inflammatory etiologies may manifest with acute onset, systemic symptoms, or associated autoimmune conditions [**12**].

Primary lacrimal gland lymphoma refers to lymphomas that originate within the lacrimal gland itself. On the other hand, systemic lymphomas, such as non-Hodgkin lymphoma, can involve the lacrimal gland as a secondary manifestation.

Inflammatory conditions such as Sjögren’s syndrome can mimic lacrimal gland lymphoma. These conditions may present dry eyes, dry mouth, and other systemic symptoms.

Benign tumors such as pleomorphic adenomas and cysts can also present as lacrimal gland swellings. These lesions are typically painless and may have a slower growth rate compared to malignant lymphomas.

Histopathological examination remains the gold standard for definitive diagnosis of lacrimal gland lymphomas, with key features including architectural effacement, monomorphic lymphoid infiltrate, and immunohistochemical staining patterns characteristic of B-cell or T-cell lymphomas [**1**,**5**,**7**]. Differential diagnosis between lacrimal gland lymphomas and other orbital malignancies, such as adenoid cystic carcinoma or metastatic tumors, relies on careful assessment of cytological features, immunophenotypic markers, and molecular studies to delineate the specific tumor lineage and subtype [**5**,**6**].

Radiation therapy, often administered alone or in combination with chemotherapy, represents a primary treatment modality for both primary and recurrent lacrimal gland lymphomas, offering favorable outcomes in terms of disease control [**4**,**6**,**10**]. Localized radiation therapy is often preferred for cases characterized by limited disease involvement, aiming to preserve ocular function while achieving optimal disease control and minimizing treatment-related toxicity [**10**]. Radiotherapy for lacrimal gland lymphomas has evolved, with modern techniques allowing for precise targeting of tumor tissue while minimizing radiation-related toxicities, thereby improving treatment tolerability and long-term ocular function [**10**].

Surgical intervention remains a cornerstone in lacrimal gland lymphomas management, aiming to achieve complete resection of localized tumors when feasible [**11**]. In select cases with extensive local invasion or refractory disease, orbital exenteration may be considered a salvage option, although its utilization remains infrequent [**13**]. In the case of our patient, considering the corneal damage due to exposure from marked chemosis and ineffective eyelid closure, we decided to perform the surgical intervention primarily, both for diagnostic and curative purposes.

Chemotherapy combinations, such as CHOP (cyclophosphamide, doxorubicin, vincristine, and prednisone) or rituximab plus CHOP (R-CHOP), have demonstrated efficacy in inducing remission and improving survival rates in cases of advanced or disseminated disease [**4**,**8**].

Rituximab, a monoclonal antibody targeting CD20, has emerged as a valuable therapeutic agent in the treatment of B-cell lymphomas affecting the lacrimal gland, either as monotherapy or in combination with other treatment modalities [**4**,**10**]. This targeted therapy has revolutionized the treatment landscape for lacrimal gland lymphomas, offering promising results in terms of response rates and long-term outcomes [**4**,**10**].

Multimodal therapy, including surgery, radiation therapy, and chemotherapy, has become increasingly utilized, leading to more effective disease control and improved survival rates [**4**,**6**,**10**].

Close post-treatment surveillance is imperative for monitoring treatment response, detecting disease relapse, and managing potential late effects of therapy, ensuring optimal long-term outcomes for patients with lacrimal gland lymphomas [**2**,**3**].

Prognostic factors influencing the clinical course and outcomes of lacrimal gland lymphomas include tumor histology, stage at presentation, presence of systemic involvement, and molecular characteristics [**5**,**8**].

While localized disease often carries a favorable prognosis with high rates of disease-free survival, advanced or disseminated disease poses greater challenges and may be associated with poorer outcomes despite aggressive treatment approaches [**5**].

## Conclusions

The management of lacrimal gland lymphomas requires a multidisciplinary approach involving ophthalmologists, oncologists, and radiation therapists. Treatment decisions are guided by tumor histology, disease stage, and patient-specific factors. Advancements in diagnostic techniques and therapeutic modalities have improved treatment outcomes over time. Despite these advancements, the rarity and heterogeneity of lacrimal gland lymphomas necessitate individualized treatment strategies and close monitoring for disease recurrence. Collaborative efforts, including international multicenter studies and cancer registries, have enhanced our understanding of epidemiology, treatment patterns, and outcomes, guiding clinical decision-making and research initiatives. Close collaboration among specialists is crucial for accurate diagnosis and optimal management of lacrimal gland lesions.

**Conflict of Interest Statement**

The authors state no conflict of interest.

**Informed Consent and Human and Animal Rights Statement**

Informed consent has been obtained from the individual included in this study.

**Authorization for the use of human subjects**

Ethical approval: The research related to human use complies with all the relevant national regulations, and institutional policies, as per the tenets of the Helsinki Declaration, and has been approved by the review board of CF2 Clinical Hospital, Bucharest, Romania (8933/13.06.2024).

**Acknowledgments**

None.

**Sources of Funding**

None.

**Disclosures**

None.
